# Oral mucositis after tacrolimus/sirolimus or cyclosporine/methotrexate as graft‐versus‐host disease prophylaxis

**DOI:** 10.1111/odi.13663

**Published:** 2020-10-27

**Authors:** Karin Garming Legert, Olle Ringdén, Mats Remberger, Johan Törlén, Jonas Mattsson, Göran Dahllöf

**Affiliations:** ^1^ Department of Dental Medicine Karolinska Institutet Huddinge Sweden; ^2^ Department of Clinical Sciences, Intervention and Technology Translational cell therapy research group Karolinska Institutet Stockholm Sweden; ^3^ Department of Medical Sciences Uppsala University and KFUE Uppsala University Hospital Uppsala Sweden; ^4^ Cell Therapy and Allogeneic Stem Cell Transplantation (CAST) Karolinska University Hospital Stockholm Sweden; ^5^ Department of Oncology‐Pathology Karolinska Institutet Stockholm Sweden; ^6^ Gloria and Seymour Epstein Chair in Cell Therapy and Transplantation University of Toronto Toronto Canada; ^7^ Princess Margaret Cancer Centre University of Toronto Toronto Canada; ^8^ Center for Oral Health Services and Research Trondheim Norway

**Keywords:** dental, graft‐versus‐host disease, haematopoietic stem cell transplantation, longitudinal, mucosa

## Abstract

**Objectives:**

To determine whether treatment with tacrolimus plus sirolimus (Tac/Sir) as a prophylaxis for graft‐versus‐host disease worsens severe oral mucositis and delays healing compared to cyclosporine plus methotrexate (CsA/Mtx) following haematopoietic stem cell transplantation.

**Subjects and Methods:**

The study comprised 141 patients: 73 randomized to receive Tac/Sir and 68 to receive CsA/Mtx. The oral mucositis assessment scale and toxicity grading according to WHO were used to assess the severity, peak and duration of oral mucositis from the day −3 to day 24 post‐transplant.

**Results:**

Eighty‐seven patients developed oral mucositis in the first 24 days post‐transplant. No significant difference in oral mucositis severity between the Tac/Sir and CsA/Mtx groups was observed. The peak oral mucositis score occurred on day 10 in both groups. Although oral mucositis scores had returned to baseline in the CsA/Mtx group on day 24 post‐transplant, no significant difference compared with the Tac/Sir group was found.

**Conclusions:**

The introduction of tacrolimus/sirolimus as a graft‐versus‐host disease prophylaxis in haematopoietic stem cell transplantation increased neither the incidence nor severity of oral mucositis compared with cyclosporine/methotrexate. Furthermore, oral mucositis healing was not prolonged and followed the same time pattern as cyclosporine/methotrexate.

## 1 INTRODUCTION

Allogeneic haematopoietic stem cell transplantation (HSCT) is a curative treatment for otherwise lethal haematopoietic disorders (Goldstone & Rowe, 2009; Negrin, 2014; Sureda et al., 2015). Continual advancement in transplant procedures has steadily improved patient outcome over time, but graft‐versus‐host disease (GVHD) remains a serious complication of HSCT. Both acute and chronic GVHD contribute significantly to morbidity and mortality after treatment (Deeg, 2007; Gooley et al., 2010; Remberger et al., 2011; Ziakas et al., 2014).

Oral mucositis (OM) is a frequent and sometimes severe side effect of post‐transplant conditioning therapy that refers to painful mucosal ulcerations of the mouth and gastro‐intestinal tract (Garming Legert et al., 2014). Between 75% and 100% of HSCT recipients develop OM (Elad et al., 2015). Direct cell injury mediated by chemotherapy and radiation characterize OM. Cell injury is a consequence of a complex cascade beginning with cell death and the release of reactive oxygen species, progression through a series of steps in which biological pathways are activated and amplified, and culminating in mucosal ulcerations (Villa & Sonis, 2015).

Since the 1980s, a combination of cyclosporine and methotrexate (CsA/Mtx) has reduced severe acute GVHD (aGVHD) and improved treatment‐related mortality after HSCT (Reshef, 2012; Ringdén et al., 1993; Storb et al., 1989). In the last decade, a regimen of tacrolimus and sirolimus (Tac/Sir) has shown promising immunosuppressive capacity and other desirable properties in solid organ transplantation (Cutler et al., 2004, 2007). Sirolimus binds to the mammalian target of rapamycin (mTOR) in T cells and suppresses T‐cell proliferation by inhibiting progression from the G1 to the S phase of the cell cycle (Li et al., 2014). Sirolimus has anti‐proliferative effects on fibroblasts, endothelial cells and smooth muscle cells (Akselband et al., 1991), suggesting impaired mucosal healing (Macdonald, 2007). Recently, studies on sirolimus in HSCT have found a decreased incidence of aGVHD and treatment‐related toxicity at the expense of higher rates of transplant‐associated thrombotic microangiopathy and related endothelial injury syndromes (Cutler et al., 2014; Shayani et al., 2013). Aside from HSCT, a high incidence of painful oral ulcers has been reported in the kidney transplant setting (van Gelder et al., 2003).

These efforts to address GVHD, including new combinations of immunosuppressive drugs and new immunosuppressive strategies, confer a risk of new side effects. One risk is the development of oral lesions in patients subjected to HSCT; another is that healing of OM lesions may be prolonged. In severely neutropenic patients, higher risks of generalized infections and prolonged hospital stays are possibilities (Garming Legert et al., 2014; Sonis et al., 2001).

Our group has previously published the results of a prospective randomized trial comparing the immunosuppressive regimens of Tac/Sir with CsA/Mtx after HSCT (Törlén et al., 2016). In that trial, no significant differences could be demonstrated between the two prophylactic regimens when comparing the cumulative incidence of aGVHD with transplant‐related mortality.

The aim of the present study, a substudy of the previous clinical trial, was to determine whether treatment with Tac/Sir as prophylaxis for GVHD worsens severe OM and delays healing compared to the standard regimen of CsA/Mtx.

## 2 MATERIALS & METHODS

### 2.1 Patients

In the main clinical trial, patients were randomized in a prospective, open‐label, phase III, multicentre trial comparing Tac/Sir and CsA/Mtx as GVHD prophylaxis in the setting of HSCT; they were enrolled at one centre in Stockholm, Sweden (September 2007 to January 2014), and one centre in Turku, Finland (January 2010 to December 2011). The Regional Board of Ethics in Stockholm (DNR [Daybook no.] 2006/1430‐31/3) and the Swedish Medical Products Agency (DNR 151:2007/38987) approved the study protocol, which was then registered with ClinicalTrials.gov (#NCT00993343) and the European Clinical Trials Database (EudraCT, #2006‐006577‐25). The study followed the guidelines of the Declaration of Helsinki. All authors attest the accuracy of the reported study data and adherence to the study protocol.

The primary endpoint of the clinical trial was NIH grades II–IV aGVHD in the two treatment groups within 200 days post‐HSCT. The secondary endpoint was incidence and severity of OM in enrolled patients. All eligible participants, or their parents or guardians if the participant was under 18 years of age (see Törlén et al., 2016 for a detailed description of the inclusion and exclusion criteria), signed informed‐consent forms.

The present OM substudy comprised only the Stockholm cohort (141 patients, Table 1): 73 patients were randomized to receive Tac/Sir, and 68 were randomized to receive CsA/Mtx as prophylaxis for GVHD.

**TABLE 1:** Patient and transplant characteristics according to graft‐versus‐host disease prophylaxis treatment

Variables	CsA/Mtx (*n* = 68)	Tac/Sir (*n* = 73)	*p*‐value^a^
Age (years, range)	55 (15–71)	53 (1–68)	.41
Children (<18 years)	1	4	
Sex (Male/Female)	40/28	45/28	.86
Diagnosis			
Acute leukaemia	30	30	.85
Chronic leukaemia	6	13	.19
Lymphoma	11	10	.86
Myelodysplastic syndrome	20	15	.31
Myeloma	1	5	.24
Disease stage (early/late)	32/36	23/50	.08
MAC/RIC	15/53	17/56	.75
TBI‐based conditioning	18	27	.21
ATG	51	49	.36
FtoM	10	9	.81
Stem cell source (BM/PBSC)	5/63	8/65	.57
TNC dose × 10^8^/kg (range)	10.6 (1.8–24.5)	11.4 (1.8–42.8)	.29
CD34 + cell dose × 10^6^/kg (range)	7.5 (1.2–22.8)	6.9 (1.3–16.4)	.50
Donor (sibling/MUD/URD)	18/49/1	24/49/0	.47
Recipient virus (0−1/2−4)^b^	7/61	7/66	1.00
Donor virus (0−1/2−4)^b^	16/50	15/57	.69

Abbreviations: ATG, anti‐thymocyte globulin; BM, bone marrow; CsA/Mtx, cyclosporine/methotrexate; FtoM, Female‐to‐male transplantation; MAC, myeloablative conditioning; MUD, matched unrelated donor; PBSC, peripheral blood stem cells; RIC, reduced‐intensity conditioning; Tac/Sir, tacrolimus and sirolimus; TBI, total body irradiation; TNC, total nucleated cells; URD, unrelated donor.

^a^ Chi‐square test.

^b^ Subjects seropositive to no. of herpes virus family members.

### 2.2 Haematopoietic stem cell transplantation treatment protocols

Disease indication, age and clinical standards at the time of the transplant (at the HSCT centre at Karolinska University Hospital in Stockholm) were used to determine pretransplant conditioning regimens. Thirty‐two patients received a myeloablative protocol consisting of one of the following: (a) cyclophosphamide (Cy) 50 mg/kg/d for 4 days or (b) Cy 60 mg/kg/d for 2 days in combination with fractionated total body irradiation (TBI) with 12 Gy, given in fractions of 3 Gy over 4 days. Reduced‐intensity conditioning consisted of one of the following: (a) fludarabine 30 mg/m^2^/d for 3–6 days in combination with Cy 60 mg/kg/d for 2 days, (b) 2 × 3 Gy TBI and Cy 60 mg/kg/d for 2 days, (c) 2 Gy TBI, treosulphan 14 g/m^2^ for 3 days or (d) Bu 4 mg/kg/d for 2 days. T‐cell depletion by administration of anti‐thymocyte globulin was part of the conditioning regimen for patients receiving grafts from an unrelated donor and patients with non‐malignant disorders; doses were previously described (Törlén et al., 2016). Stem cell source was peripheral blood progenitor cells or bone marrow. Supportive care followed previously published institutional standards (Forslöw et al., 2006).

**FIGURE 1:**
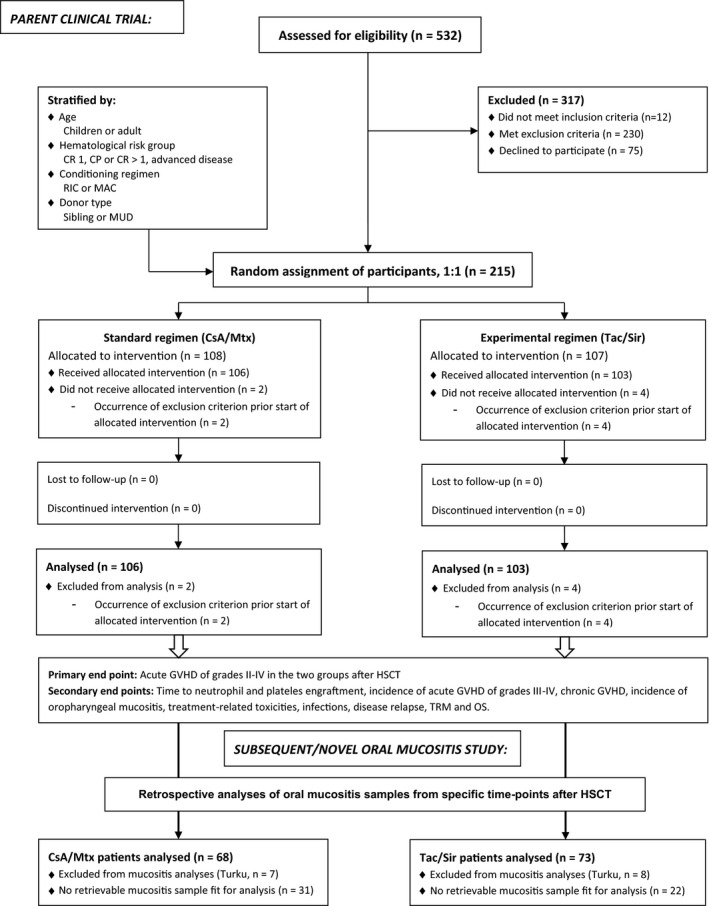
Flow of patients in the parent randomized clinical trial, and subsequent laboratory analyses in the present substudy

GVHD prophylaxis was assigned by randomization and consisted of Tac/Sir or CsA/Mtx. Randomization occurred 4 to 7 days before HSCT graft infusion and was performed at a ratio of 1:1 with the use of random block sizes, stratified by age (children or adult), haematological risk group (CR 1, CP or >CR 1, advanced disease), conditioning regimen (reduced‐intensity conditioning [RIC] or myeloablative conditioning [MAC]) and donor type (sibling or matched unrelated donor [MUD]). Patients with a non‐malignant disease were included in the low haematological risk group. No blinding was attempted after randomization (Figure 1). Patients given Tac/Sir started their prophylaxis in combination on day −3 before graft infusion. Treatment comprised (a) Sir with a bolus dose of 6 mg/kg in adults and 0.1 mg/kg in children, followed by continuous adjustment to reach target levels of 3–12 ng/ml and (b) 0.15 mg kg^−1^ day^−1^ Tac. For all diagnoses, patients in the CsA/Mtx group started CsA on day −1 and Mtx 15 mg/m^2^ on day + 1, with consecutive doses of 10 mg/m^2^ on days + 3, +6 and +11. Following HSCT, both regimens were discontinued after tapering 3 to 6 months, depending on grafts and in the absence of GVHD.

### 2.3 Assessment of acute GVHD

The attending physicians diagnosed aGVHD clinically and assigned a grade from 0 to IV using previously published criteria (Przepiorka et al., 1995). Biopsies from the skin, gut and liver were assessed according to the routines at the centre.

### 2.4 Oral examinations

Baseline examination of the oral cavity included a radiographic and a clinical examination approximately 14 days before HSCT, before the start of conditioning treatment. If needed, infectious foci in the oral cavity were treated conservatively. The nurses were trained and calibrated to diagnose OM according to the International Classification of Diseases from the World Health Organization (World Health Organization, 1979). Post‐treatment, the nurses conducted oral examinations of the patients daily, from 3 days before the transplantation, until day 24 after HSCT or discharge from the hospital (*n* = 141). The patients self‐reported pain in the oral cavity on a visual analogue scale. A dentist visited the patients 3 days a week to examine the oral mucosa and record the clinical features of OM using the oral mucositis assessment scale (OMAS; Sonis et al., 1999) and the World Health Organization Oral Toxicity Scale Grading of Oral Mucositis (Sonis et al., 2001). There was a good correlation between the WHO OM grading scale and the OMAS (r = 0.74, *p* < .001).

### 2.5 Standard oral hygiene protocol

The standard oral hygiene protocol included careful tooth brushing twice a day with interdental cleaning. All patients were instructed to suck on ice chips throughout conditioning, up to the day of transplantation, and especially during chemotherapy. Patients with symptoms of OM were recommended to continue sucking ice chips as much as possible. They were also instructed to rinse their mouth with saline solution once every waking hour—from transplantation until the neutrophil blood cell count exceeded 0.5 x 10^9^/litre. Patients with symptoms of dry mouth received a saliva substitution or lubricants.

### 2.6 Statistical analyses

In comparisons between the Sir/Tac and CsA/Mtx groups, variables from the WHO grading scale were used. Categorical variables were compared with the chi‐square method and continuous variables with the Mann–Whitney U test. Factors with a *p* < .20 in the univariate analysis (Table 2) were included in the backwards elimination multivariate analysis. Multivariate analyses for the dichotomous OM variable (grades 0–1 versus grades 2–4) were performed with the logistic regression method. OM was dichotomized as grade 0–1 versus 2–4, since grades 2–4 are considered clinically significant and may cause patient suffering. Analyses were performed using Statistica software (Statsoft, Tulsa, OK, USA).

**TABLE 2:** Patient and transplant characteristics dichotomized according to the WHO oral mucositis grade (OM)

Variables	OM 0–1 (*n* = 54)	OM 2–4 (*n* = 87)	*p*‐value^a^
Age (years, range)	54 (1–70)	53 (14–71)	.60
Children (<18 years)	3	2	.37
Sex (male/female)	40/14	45/42	**.013**
Diagnosis			
Acute leukaemia	16	44	**.022**
Chronic leukaemia	8	11	.91
Lymphoma	11	10	.23
Myelodysplastic syndrome	15	20	.66
Myeloma	4	2	.30
Disease stage (early/late)	16/38	39/48	.11
MAC/RIC	4/50	28/59	**<.001**
TBI‐based conditioning	18	27	.85
ATG	39	61	.85
FtoM	8	11	.80
SC source (BM/PBSC)	5/49	8/79	1.00
TNC dose ×10^8^/kg (range)	11.2 (2.5–36.4)	11.1 (1.8–42.8)	.90
CD34 + cell dose ×10^6^/kg (range)	6.9 (1.2–15.0)	7.6 (1.8–22.8)	.26
Donor (sibling/MUD/URD)	15/39/0	27/59/0	.71
Recipient virus (0–1/2–4)^b^	6/48	8/79	.78
Donor virus (0–1/2–4)^b^	12/41	19/66	1.00

Abbreviations: ATG, anti‐thymocyte globulin; BM, bone marrow; FtoM, female‐to‐male transplantation; MAC, myeloablative conditioning; MUD, matched unrelated donor; PBSC, peripheral blood stem cells; RIC, reduced‐intensity conditioning; TBI, total body irradiation; TNC, total nucleated cells; URD, unrelated donor.

^a^ Chi‐square test.

^b^ Subjects seropositive to no. of herpes virus family members, 0‐1/2‐4.

## 3 RESULTS

### 3.1 Patient characteristics

Table 1 lists the characteristics of all study participants. Assessments of OM with the WHO oral toxicity grading criteria were made in 68 patients in the Mtx/CsA group and 73 in the Tac/Sir group. No significant between‐group differences were observed in age, diagnosis of or indication for HSCT, intensity of conditioning regimens or donor types.

### 3.2 Assessment of oral mucositis

During the first 24 days post‐transplant, 54 patients received an OM diagnosis of WHO grade 0–1 and 87 patients, of WHO grade 2–4; when regrouped, 41 patients had an OM diagnosis of WHO grade 3–4. In the univariate analysis, female patients (*p* = .013), patients with acute leukaemia (*p *= .022) and patients who had undergone myeloablative conditioning (*p* < .001) were significantly more often diagnosed with OM grade 2–4 than other patients (Table 2). OM severity (i.e. patients with WHO grades 0–1 versus 2–4) differed non‐significantly between the two GVHD prophylaxis treatment groups (*p *= .49, Table 3). On day 10, median OM in the CsA/Mtx group was 2.0 and mean OM 1.7 (*SD* 1.30); in the Tac/Sir group, median OM was 2.0 and mean OM 1.6 (*SD* 1.23; *p *= .59). Maximum OM grade was a median of 2.0 in the CsA/Mtx group and a mean of 1.86 (CD 1.22); in the Tac/Sir group, the maximum OM grade was a median was 2.0 and a mean of 1.75 (*SD* 1.19; *p *= .57). In both groups, the mean duration of neutropenia was 17 days (*p *= .61).

**TABLE 3:** Incidence of graft‐versus‐host disease (GVHD) in patients who had undergone haematopoietic stem cell transplantation, dichotomized according to the WHO oral mucositis grade (OM)

Variables	OM 0–1 (*n* = 54)	OM 2–4 (*n* = 87)	*p*‐value^a^
GVHD prophylaxis group			
Cyclosporine/Methotrexate	24	44	.49
Tacrolimus/Sirolimus	30	43	
Acute GVHD, grade			
0	18	25	.71
I	8	19	
II	22	32	
III–IV	6	11	

^a^ Chi‐square test.

There was no significant difference in incidence or grade of aGVHD between the two OM groups. No study patient was diagnosed with oral aGVHD during the period of OM grading (data not shown).

A multivariate analysis of risk factors for WHO grades 2–4 found that reduced‐intensity conditioning was associated with a significantly lower grade (odds ratio [OR] 0.18; 95% confidence interval [CI]: 0.06–0.56; *p* = .003) and female sex, with a significantly higher risk (OR 2.50; 95% CI: 1.15–5.42; *p *= .019).

A complete record of the WHO and OMAS grading from day −3 until day 24 post‐transplant was retrieved for 33 patients in the Tac/Sir group and 30 patients in the CsA/Mtx group. Both groups recorded a peak OM grade on day 10 distributed as follows: OM 0, 27 patients; OM 1, 25 patients; OM 2, 48 patients; OM 3, 30 patients; and OM 4, 11 patients. The median peak OM grade according to the WHO toxicity scale was 1.7 (range 0–4).

**FIGURE 2:**
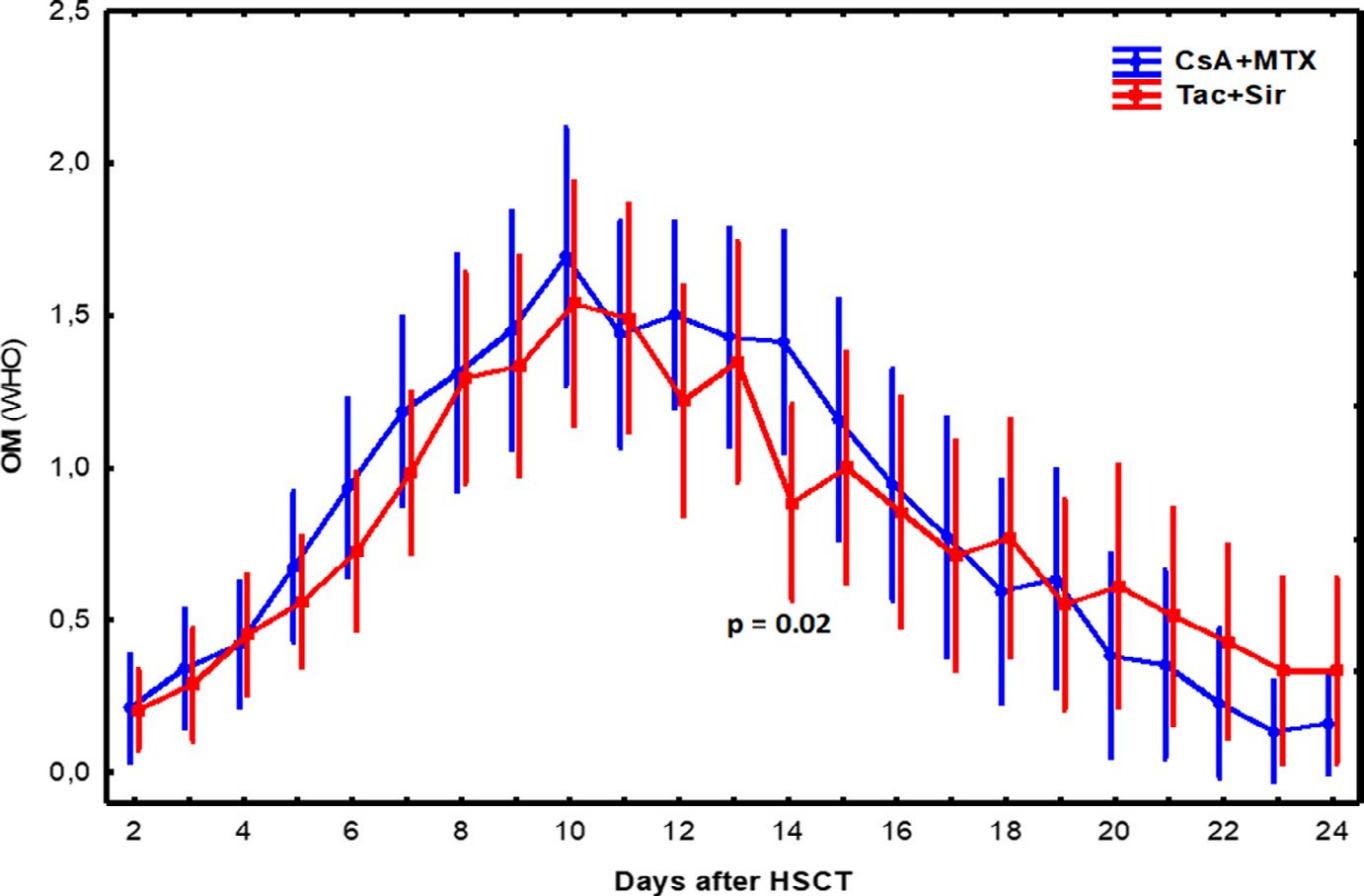
Mean severity of WHO oral mucositis grade (OM) from day of transplantation until day 24 post‐transplant in patients who had undergone haematopoietic stem cell transplantation (HSCT). Patients were treated with either cyclosporine/methotrexate (CsA/Mtx) or tacrolimus/sirolimus (Tac/Sir)

The only significant difference between the two immunosuppressive regimens occurred on day 14 post‐HSCT, where CsA/Mtx patients scored higher than Tac/Sir patients (*p *= .02). Although OM scores had returned to baseline in the CsA/Mtx group on day 24 post‐transplant, no significant difference compared with the Tac/Sir group was found (Figure 2).

Four of 141 patients (3%) developed typical oral ulcers, most likely caused by treatment with sirolimus, referred to as mTORI‐associated stomatitis (mIAS). None of these patients had previously developed oral mucositis. The lesions had an aphtous‐like appearance and developed a median of 40 days after HSCT. The ulcers tested negatively for herpes virus and were located on the lower lip (*n* = 2) and on the lateral part of the tongue (*n* = 2) and healed after sirolimus treatment ceased.

## 4 DISCUSSION

The present study found that, as a GVHD prophylaxis of OM, Tac/Sir following HSCT increased neither the incidence nor severity of OM compared with CsA/Mtx. Furthermore, OM healing was not prolonged and followed the same time pattern as CsA/Mtx. In all, 62% of the patients who underwent HSCT were diagnosed with OM, and MAC was an important risk factor. In a study on a similar HSCT population, Shouval et al. (2019) found that the incidence of moderate‐to‐severe OM was as high as 83% and depended primarily on conditioning intensity and GVHD prophylaxis. The literature describes several other risk factors in the HSCT setting, often with contradictory clinical results (Barasch & Peterson, 2003).

OM (WHO grades 2–4) was diagnosed in 58% of patients receiving Tac/Sir compared to 64% receiving CsA/Mtx, a non‐significant difference.

The present study found that the peak incidence of OM occurred on day 10 after HSCT and that the WHO OM score had returned to baseline on day 24. This corresponds to previous studies reporting a peak after 10–11 days and complete healing after 21–23 days (Cutler et al. 2005; Garming Legert et al., 2014). Despite the previously published risks of impaired wound healing in patients treated with sirolimus, we found no differences in the course of OM after HSCT. The courses of healing in those receiving Tac/Sir and those receiving CsA/Mtx were similar; the OMAS score had returned to baseline after 24 days. This is in contrast to Cutler et al. (2014), who found more rapid healing of oropharyngeal mucositis in the Tac/Sir group.

Wound‐healing complications associated with sirolimus therapy are typically diagnosed within the first few months following transplant (Troppmann et al., 2003). In the kidney transplant setting, because of low nephrotoxicity, the use of sirolimus in combination with a calcineurin inhibitor has resulted in a low incidence of graft rejection, a lowered occurrence of new malignancies and a possibility of steroid withdrawal among high immune responder kidney transplant recipients (Kahan, 2000). At the same time, the use of sirolimus has been associated with increased wound‐healing complications, defined as lymphocele, wound infection and incisional hernia (Knight et al., 2007).

Based on the different mechanisms of action between calcineurin inhibitors and sirolimus, an impaired wound‐healing process of OM could be anticipated when sirolimus is used in HSCT. In the present study, the target plasma concentration for sirolimus was held lower (3–12 ng/ml) than the recommended plasma concentration in organ transplantation (for kidney transplants: 10–15 ng/ml in the early phase, 8–12 ng/ml in the intermediate phase, and in absence of graft rejection, 6–10 ng/ml in later phases). This could explain the absence of more severe OM scores and a prolonged healing phase. On the other hand, Troppmann et al. (2003) found that the development of wound‐healing complications is not necessarily a concentration‐dependent effect. Moreover, in the present study, sirolimus was discontinued earlier (after a median of 68 days treatment days) compared to the much longer treatment schemes in organ transplant settings.

We have previously shown a high correlation between the WHO toxicity score and the OMAS (Garming Legert et al., 2014), although they were developed for different purposes. Risk factors for OM in the multivariate analysis were myeloablative therapy and female sex. In the present study, risk factors for OM in the multivariate analysis were myeloablative therapy and female sex. The 2014 study (Garming Legert et al., 2014) was done on a larger cohort than in the present study and found, in addition to these two risk factors, that female‐to‐male transplantation and being seropositive for 3–4 different herpes viruses were also risk factors. The significantly increased risk of mucositis in patients treated with MAC as opposed to RIC is in line with a previous prospective randomized study in HSCT patients with myeloid malignancies (Ringdén et al., 2013). Patients randomized to MAC in the present study had a significantly higher median mucositis grade (OM 4) than RIC patients (OM 1). Several other toxicities were also increased in the MAC group.

Three per cent of patients treated with Tac/Sir developed mIAS induced by the Tac/Sir therapy with a median onset of 40 days post‐transplant. This result is similar to the Villa et al. (2015) study, which reported mIAS in 2% of HSCT recipients 55 days post‐transplant. The clinical appearance of mIAS was similar to the observations of Peterson et al. (2016) regarding both size and involvement of non‐keratinized oral mucosa.

The strengths of this study include an RCT study design and randomization of the participants. Other strengths include the large size of the cohort of patients who underwent HSCT and the more than 3 weeks of continuous follow‐up with oral examinations. Limitations include a lack of stratification of patients during randomization concerning level of HLA match, which is a known risk factor of OM in HSCT settings. No blinding was attempted after randomization. OM was analysed using the WHO toxicity scale, although the OMAS scale is more often used in research. The two scales, however, have good correlation.

In conclusion, the present study found that, as a GVHD prophylaxis, tacrolimus/sirolimus following HSCT increased neither the incidence nor severity of OM compared with cyclosporine/methotrexate. Furthermore, we show that OM healing was not prolonged and followed the same time pattern as cyclosporine/methotrexate.

## CONFLICTS OF INTERESTS

The authors have no conflicts of interests to declare.

## AUTHOR CONTRIBUTION

**Karin U E Garming Legert:** Conceptualization; Data curation; Investigation; Methodology; Project administration; Resources; Supervision; Validation; Visualization; Writing‐original draft; Writing‐review & editing. **Olle Ringdén:** Conceptualization; Data curation; Funding acquisition; Methodology; Project administration; Resources; Supervision; Visualization; Writing‐review & editing. **Mats Remberger:** Data curation; Formal analysis; Software; Validation; Writing‐review & editing. **Johan Törlen:** Conceptualization; Data curation; Validation; Visualization; Writing‐review & editing. **Jonas Mattsson:** Conceptualization; Data curation; Funding acquisition; Methodology; Resources; Validation; Writing‐review & editing. **Göran Dahllöf:** Conceptualization; Validation; Visualization; Writing‐original draft; Writing‐review & editing.

### PEER REVIEW

The peer review history for this article is available at https://publons.com/publon/10.1111/odi.13663.
